# Early Diagnosis of Hereditary Angioedema in Japan Based on a US Medical Dataset: Algorithm Development and Validation

**DOI:** 10.2196/59858

**Published:** 2024-09-13

**Authors:** Kouhei Yamashita, Yuji Nomoto, Tomoya Hirose, Akira Yutani, Akira Okada, Nayu Watanabe, Ken Suzuki, Munenori Senzaki, Tomohiro Kuroda

**Affiliations:** 1 Department of Hematology and Oncology Graduate School of Medicine Kyoto University Kyoto Japan; 2 Department of Palliative Care Medicine Niigata City General Hospital Niigata Japan; 3 Department of Traumatology and Acute Critical Medicine Graduate School of Medicine Osaka University Osaka Japan; 4 Division of Medical Information Technology and Administration Planning Kyoto University Hospital Kyoto Japan; 5 Healthcare and Life Science, IBM Consulting IBM Japan, Ltd Tokyo Japan

**Keywords:** machine learning, screening, AI, prediction, rare diseases, HAE, electronic medical record, real world data, big data, angioedema, edema, ML, artificial intelligence, algorithm, algorithms, predictive model, predictive models, predictive analytics, predictive system, practical model, practical models, early warning, early detection, real world data, RWD, Electronic health record, EHR, electronic health records, EHRs, EMR, electronic medical records, EMRs, patient record, patient record, health record, health records, personal health record, PHR

## Abstract

**Background:**

Hereditary angioedema (HAE), a rare genetic disease, induces acute attacks of swelling in various regions of the body. Its prevalence is estimated to be 1 in 50,000 people, with no reported bias among different ethnic groups. However, considering the estimated prevalence, the number of patients in Japan diagnosed with HAE remains approximately 1 in 250,000, which means that only 20% of potential HAE cases are identified.

**Objective:**

This study aimed to develop an artificial intelligence (AI) model that can detect patients with suspected HAE using medical history data (medical claims, prescriptions, and electronic medical records [EMRs]) in the United States. We also aimed to validate the detection performance of the model for HAE cases using the Japanese dataset.

**Methods:**

The HAE patient and control groups were identified using the US claims and EMR datasets. We analyzed the characteristics of the diagnostic history of patients with HAE and developed an AI model to predict the probability of HAE based on a generalized linear model and bootstrap method. The model was then applied to the EMR data of the Kyoto University Hospital to verify its applicability to the Japanese dataset.

**Results:**

Precision and sensitivity were measured to validate the model performance. Using the comprehensive US dataset, the precision score was 2% in the initial model development step. Our model can screen out suspected patients, where 1 in 50 of these patients have HAE. In addition, in the validation step with Japanese EMR data, the precision score was 23.6%, which exceeded our expectations. We achieved a sensitivity score of 61.5% for the US dataset and 37.6% for the validation exercise using data from a single Japanese hospital. Overall, our model could predict patients with typical HAE symptoms.

**Conclusions:**

This study indicates that our AI model can detect HAE in patients with typical symptoms and is effective in Japanese data. However, further prospective clinical studies are required to investigate whether this model can be used to diagnose HAE.

## Introduction

The rare genetic disease hereditary angioedema (HAE) induces acute attacks of swelling in various regions of the body, including the face, hands, arms, legs, abdomen, genitals, buttocks, and throat. Gastrointestinal disturbances such as abdominal pain, nausea, and vomiting are frequently associated with edema. Laryngeal edema is rare, even though more than half of the patients with HAE encounter this life-threatening condition [1]. Its global prevalence is estimated to be 1 in 50,000 people, with no reported bias among different ethnic groups [2]. In Japan, about 1 in 250,000 people are diagnosed with HAE, which suggests that only 20% of potential HAE cases are identified [3], suggesting that many patients with HAE remain undiagnosed in Japan. Furthermore, in Japan, the mean duration from the first symptoms to diagnosis is 15.6 years [4], which is longer than that in Europe and the United States [5,6]. Early detection of undiagnosed patients is critical for effective treatment of HAE.

To overcome this situation in Japan, the Diagnostic Consortium to Advance the Ecosystem for Hereditary Angioedema (DISCOVERY) was established in 2021 [7]; it aimed to identify patients with undiagnosed HAE and provide them with appropriate treatment as early as possible.

In this study, we aimed to develop an artificial intelligence (AI) model that can detect suspected patients with HAE using medical history data (claims and electronic medical records [EMRs]) in the United States. We then sought to validate the model’s performance in detecting HAE cases. In addition, we conducted a pilot study at Kyoto University Hospital (KUHP) using the EMR data to verify the model’s applicability to medical data obtained from the Japanese population. The main objective of this study was to verify whether this model could identify patients with a history of HAE or related diseases.

## Methods

### Overview

First, we developed an AI model using medical history data from the United States as a reference. Thereafter, we applied the model to medical history data from Japan and verified its efficacy using a Japanese dataset. Note that we used a large dataset of patients from the United States as input for the model, considering that HAE is a rare disease.

### Initial Model Development with US Dataset

#### Data Selection

The Merative MarketScan Explorys Claims-EMR Data Set (formerly IBM Watson Health) [8] was used to obtain patient-level linked claims and EMR data for US patients. The diagnoses and prescription histories of patients with edema or digestive symptoms from January 2012 to January 2021 were identified from the dataset and were used to build our model. Data from a total of 4,283,815 patients were used in the study.

To identify the diagnosis history of patients, the *International Classification of Diseases* (*ICD*) [9] code (ninth and 10th edition) available in this dataset was used. However, the *ICD* code for HAE (D84.1) represents “defects in the complement system,” which is also applicable to other similar diseases. Therefore, we used the prescription history of drugs administered only for HAE (Table 1) to distinguish patients with HAE. We categorized the patients with a prescription history of these drugs as the “HAE group,” representing patients presumed to have HAE.

**Table 1:** US Food and Drug Administration–approved medications used only for hereditary angioedema (as of January 2022).

Proprietary name	Nonproprietary name	Product NDC^a^
BERINERT	Human C1-esterase inhibitor	63833-825
CINRYZE	Human C1-esterase inhibitor	42227-081 42227-083
FIRAZYR	Icatibant acetate	54092-702
HAEGARDA	Human C1-esterase inhibitor	63833-828 63833-829
KALBITOR	Ecallantide	47783-101
ORLADEYO	Berotralstat hydrochloride	72769-101 72769-102
RUCONEST	C1 esterase inhibitor recombinant	70383-350 69913-350 71274-350
TAKHZYRO	lanadelumab-flyo	47783-644
Icatibant (Generic)	Icatibant acetate or Icatibant	0093-3066 24201-207 60505-6214 63323-574 68462-828 69097-664 71225-114
SAJAZIR	Icatibant	70709-013

^a^NDC: National Drug Code.

**Figure 1:**
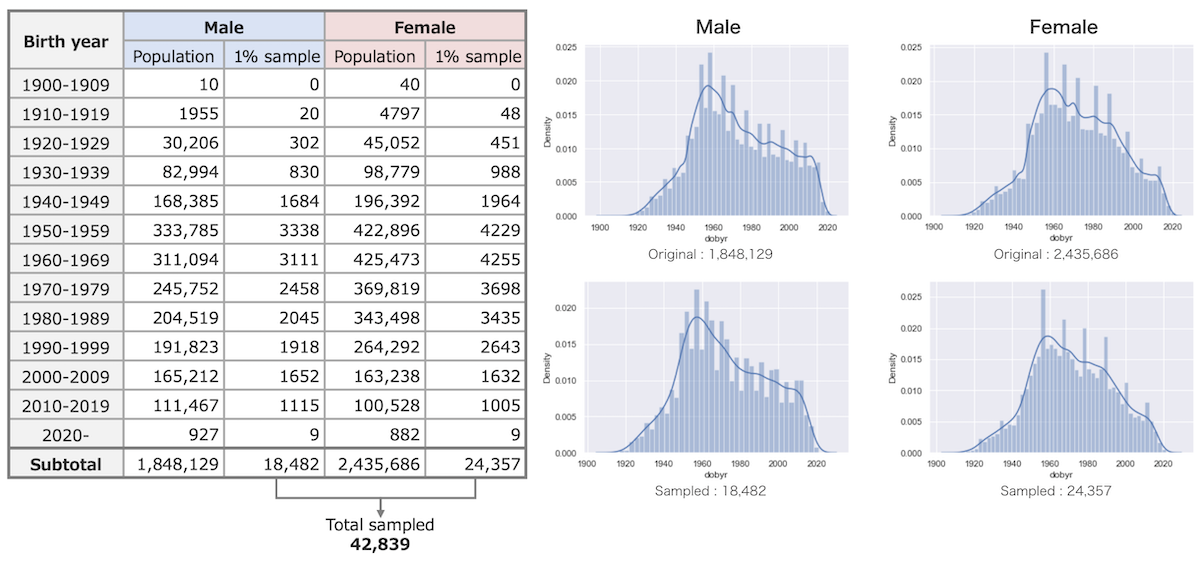
Comparison of the distribution of the 1% sampled data set with that of the population. dobyr: date of birth year.

To maintain the demographic characteristics of the original data, the control group was randomly sampled from 1% of the remaining patients, with a fixed ratio of age groups and male-to-female ratio (Figure 1). Note that this was crucial to reduce the data volume to operate the model using limited computation resources (2 central processing units and 16 GB of memory). This was done considering the potential use of the model in various medical institutions in the future.

**Figure 2:**
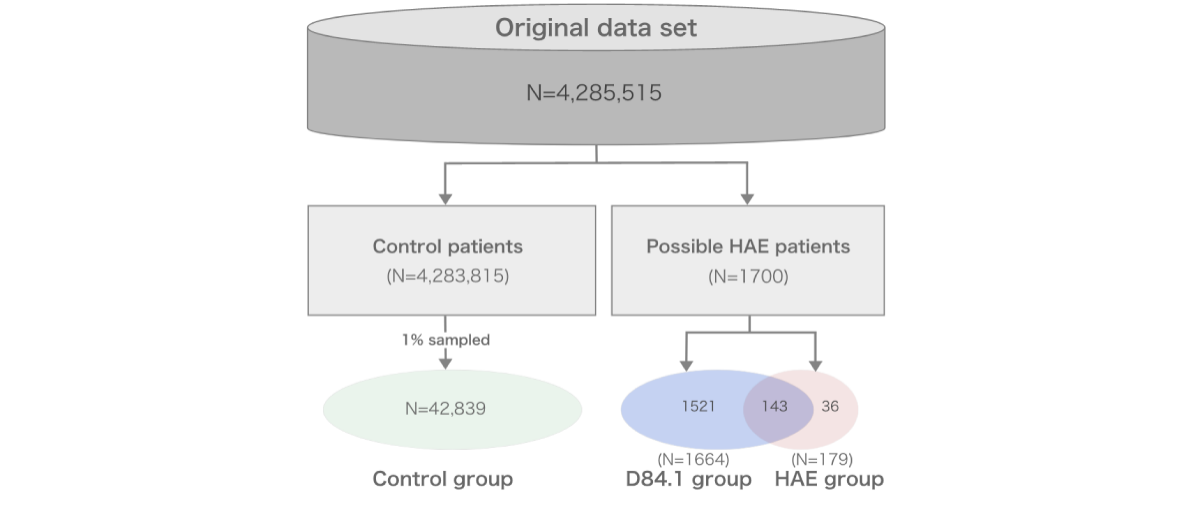
Flowchart depicting the different patient groups created using the US data set; HAE: hereditary angioedema.

Finally, 3 groups were included for model development and validation (Figure 2): the HAE group with 179 patients, D84.1 (including individuals that likely have HAE but do not have a prescription history of HAE-specific treatments) with 1521 patients, and the control group with 42,839 patients.

To develop the model, the *ICD* code was used to create features that described the diagnostic history of patients. As this dataset contained both *ICD-9* and *ICD-10* codes throughout the data period, we standardized the 2 *ICD* types. We assigned codes representing the same disease items from both *ICD-9* and *ICD-10* codes under a single ID.

#### Model Development

##### Feature Selection

We counted the number of types of *ICD* codes diagnosed in both the HAE and D84.1 groups, as these 2 groups should have similar features. Furthermore, the differences in *ICD* code types between the groups were required to create a model that can identify patients with HAE. We examined rank correlations between the 2 groups and found it to be approximately 0.08, which suggested that the 2 groups had different characteristics. We then examined specific *ICD* codes that were significantly ranked differently between the two groups and identified 25 such *ICD* codes, which were then used as the primary features in developing the model.

We also examined *ICD* codes that were diagnosed several times over a period of 1 year. This is important as patients with HAE tend to have repeated occurrences of swelling in various regions of the body [1], which can lead to the diagnosis of stomachaches and edemas. We counted the number of patients who had been diagnosed with stomachaches or edemas between 2 and 4 times per year and found a substantial difference between both groups. Considering that the medical record entry may overlap multiple times when changing the record types, we conducted the removal of duplicates based on the date and *ICD* code for each patient. Thereafter, we labeled a group of *ICD* codes related to abdominal pain or edemas and counted the number of times they were assigned in a 1-year span window for each patient based on this dataset. From this exploratory analysis, we included instances where individuals experienced four or more incidences of stomachaches and 3 or more incidences of edema per year as part of the main features of our model. The table of the explanatory variables is provided in Multimedia Appendix 1.

##### Model Building

The number of patients in the HAE group was extremely small compared with that in the control + D84.1 groups; thus, to avoid overfitting, we used bootstrap sampling [10,11] to create the model. A generalized linear model [12] with regularization terms [13,14] was adopted. We used 
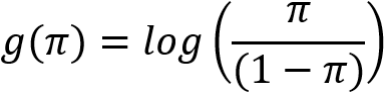
 for the link function to create a logistic model that would indicate the likelihood of the patient belonging to the HAE group. We chose logistic regression for evaluation as it allows for regression with regularization and is relatively easy to use for evaluating and interpreting feature importance by checking the coefficients. The estimation of the partial regression coefficients was calculated by the maximum likelihood method, which estimates parameters (known as maximum likelihood estimates) that maximize the likelihood of the given observed values. The regularization parameter λ was set to 1 to ensure that it was Lasso regularization.

**Figure 3:**
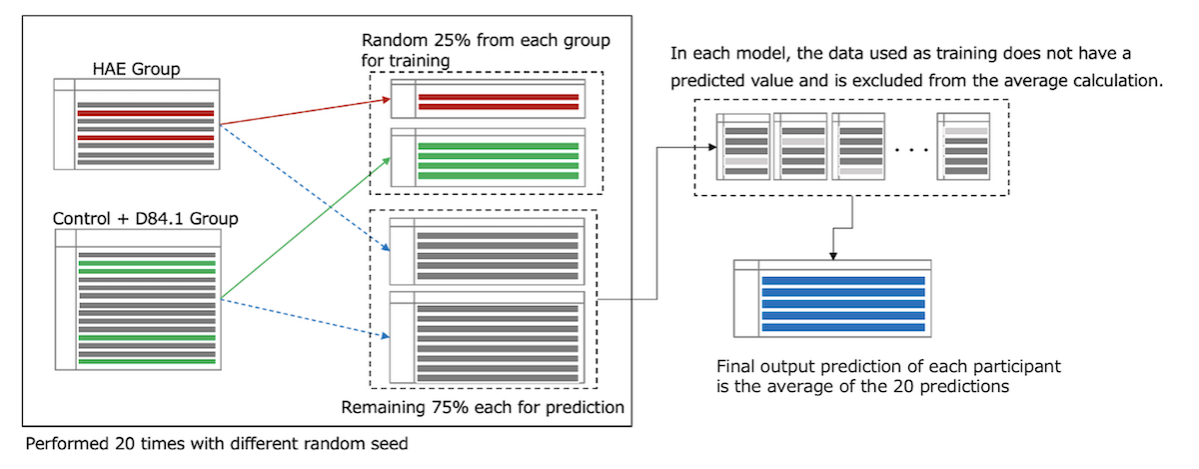
Training data extraction and prediction calculation of the constructed model. HAE: hereditary angioedema.

We used 25% of the data from the HAE group and another 25% from the control + D84.1 groups to train the model, which was then used to predict the remaining 75% of each group. This modeling process was performed 20 times with different random seeds. The average predicted value was calculated as the final output for all the patients. In each trial, the sample used as training data did not have a predicted value and was excluded from the average value calculation (Figure 3). Upon applying the regularization using Lasso regression, the number of substantial features was sorted out during each calculation by mathematically adjusting the coefficients of some variables to 0. The number of sorted features varied with an average of 10; notably, different features were selected every time.

### Evaluation Method and Threshold Setting

After obtaining the final value for each participant, we performed Welch *t* test on the 2 distributions to confirm that the 2 groups had different means. Subsequently, we defined the threshold value that yielded the most balanced classification accuracy using the receiver operating characteristic (ROC) curve. ROC curves help visualize the entire scenario of trade-offs between sensitivity and precision across a set of cutoff points. The volumes of the HAE and control + D84.1 groups were not equal; therefore, it was important to check the balance between sensitivity and precision rather than the accuracy itself.

### Model Application to Japanese EMR Data

#### Data Extraction and Model Application

**Figure 4:**
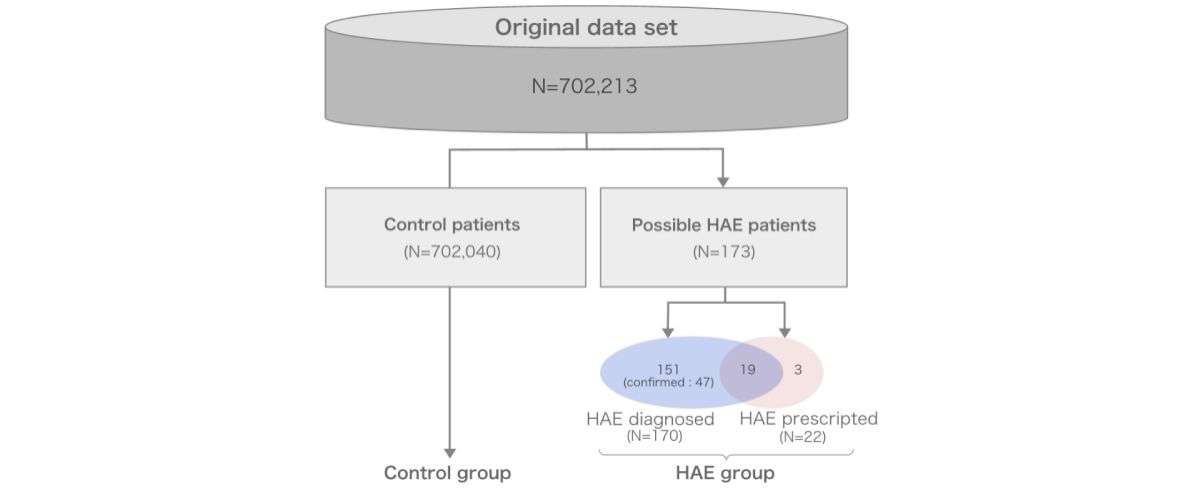
Flowchart depicting the different patient groups obtained from the KUHP data set. HAE: hereditary angioedema.

For the validation step using Japanese data, data were extracted from a data warehouse (DWH), which collects medical data from the EMR of the KUHP. Patient IDs in the DWH are pseudonymized. The medical data were obtained for a total of 702,213 patients, among which 22 had a history of HAE, 47 had a confirmed diagnosis of HAE, and 123 had a suspected diagnosis of HAE. The data for model validation included those associated with patients from all these groups (patients using drugs for HAE, patients with confirmed HAE, and patients suspected to have HAE). This was done because physicians may have suspected HAE for some patients if their symptoms were similar to those of patients with the condition. Therefore, these 3 types of patients were considered as the patients with HAE in the study (HAE group; Figure 4).

To adapt the model to Japanese data, we used the standard disease name codes widely used in Japan, as defined by the Medical Information System Development Center (MEDIS-DC) [15], instead of the *ICD* code. Although the *ICD* code is the basic classification code for diagnosis, the standard disease name codes have more subdivisions compared with the *ICD* code, and hence, they can provide a more precise clinical diagnosis. We converted the *ICD* codes using the standard disease name code master for *ICD-10* [16].

Patient data extracted from the DWH were transferred to the Google Cloud Platform server (a virtual private cloud environment) hosted at KUHP. The AI model and statistical programs were stored in a container and sent to the server. We then accessed the server through a virtual private network, which could only be accessed by the authors of this study. The model was applied to all patient data on this server.

### Ethical Considerations

This study was approved by the Ethics Committee of the Kyoto University Hospital (approval number R3750). In this study, we used pseudonymized information that had already been processed, thus individual informed consent was not required. The pseudonymized medical data is made available for academic research in accordance with KUHP’s privacy policy. Information regarding each study is publicly disclosed on the institution’s website, where patients are informed of their right to opt out along with the opt-out procedure.

## Results

### Evaluation of the Initial Model

Welch *t* test indicated that the 2 patient groups did not have the same mean values, as suggested by the *P* value of 2.2e-16. Furthermore, the area under the ROC curve was 86.4%, which was obtained when only the HAE group was set as true and all the other groups as false. The best accuracy threshold of this ROC curve was calculated as 39%, with an accuracy of 99.6%. This is because the volume of the control + D84.1 group was larger than that of the HAE group. The true-positive (sensitivity) of this threshold was only 10.6%, with a precision of 54.3%.

As we aimed to identify patients likely to have HAE, we searched for a different threshold that could improve the sensitivity while keeping the precision at an acceptable level. Considering the fact that the prediction of the HAE group had 
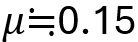
 and 
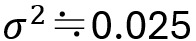
, 0.075-0.125 could be a good threshold candidate. We confirmed the sensitivity and precision for the thresholds of 0.075, 0.1, and 0.125 to determine the most balanced threshold, as shown in Table 2.

**Table 2:** Cross-tabulation was calculated at 3 different threshold values using all data groups for a detailed evaluation of different scaled precisions and group sensitivities.

		Control group			D84.1 group		D84.1 and HAE^a^		HAE group (not D84.1)		Score (%)			
		100% scale converged	1% scale							1% scale precision		100% scale precision	Sensitivity 1^b^	Sensitivity 2^c^
**Suspection statistic (threshold=0.1)**														
	Not suspected, n	4,279,500	42,795	1312		55		30		27.1		2.0	61.5	52.5
	Suspected, n	4400	44	209		88		6		—^d^		—	—	—
**Suspection statistic (threshold=0.075)**														
	Not suspected, n	4,277,900	42,779	1266		52		28		23.9		1.6	63.6	55.3
	Suspected, n	6000	60	255		91		8		—		—	—	—
**Suspection statistic (threshold=0.125)**														
	Not suspected, n	4,280,600	42,806	1343		62		33		28.5		2.4	56.6	46.9
	Suspected, n	3300	33	178		81		3		—		—	—	—

^a^HAE: hereditary angioedema.

^b^Excluding “not D84.1” patients.

^c^Including “not D84.1” patients.

^d^Not applicable.

The threshold value of 0.1 had a sensitivity of 52.5% and precision of 27.1%, indicating that 1 out of 2 known HAE group participants can be correctly detected, and 1 out of 4 detected participants should correctly belong to the HAE group. If we exclude the HAE group participants who were not diagnosed with D84.1, the sensitivity was 61.5%. This result was calculated based on 1% of the sample size of the original control group; thus, by multiplying the number of all participants from the control group by 100, we obtained a 100% scale precision of 2%. This was 2 times better than the 1% precision goal set at the beginning of the study. This means that based on this model, 1 out of 50 suspected patients is highly likely to have HAE. Considering that HAE prevalence is estimated to be 1 in 50,000 people, we can expect to find undiagnosed patients with HAE quickly and efficiently using this model output.

From a conservative standpoint, the threshold value of 0.1 seems optimal. However, to identify more potential patients with HAE, it might be better to apply the 0.075 threshold, which has a sensitivity of 55.3% and a precision of 23.9%. If we recalculate the 100% scale precision in the same manner as described above, we obtain 1.6%. This means we can still achieve our goal of 1% precision while improving the sensitivity.

In addition, we need to consider the fact that the ratio of suspected patients in the US dataset can be calculated to be approximately 0.09% with a 0.1 threshold and 0.15% with a threshold of 0.075. If this model is to be used on a much smaller volume dataset compared with the US dataset, there is an approximately 2 times higher risk of obtaining zero suspected patients with a 0.1 threshold than with the 0.075 threshold.

### Application of the Model to Japanese EMR Data

To verify the performance of this model using Japanese data, it was applied to patient data obtained from KUHP, and the output of potential patients with HAE was obtained based on the selected threshold. The diagnostic histories of these patients were stored at a single university hospital. Compared with the dataset used to build the original model, the variation and coverage of the entire diagnostic history were assumed to be relatively low. Therefore, we adopted a threshold value of 0.075 in this validation study to aggressively identify patients with HAE. We considered the HAE group (Figure 4) as the correct data for this validation.

As shown in Table 3, 65 of 173 patients with HAE were detected using this model, indicating a sensitivity of 37.6%.

**Table 3:** Cross-tabulation with precision and sensitivity scores of Kyoto University Hospital results.

		Control group	HAE^a^ group			Score (threshold=0.075)	
			Prescripted	Prescripted and diagnosed	Diagnosed	Precision (%)	Sensitivity (%)
**Suspection statistic (threshold=0.075)**						3.2	31.8
	Not suspected	701,829	2	13	93		
	Suspected	211	1	6	58		

^a^HAE: hereditary angioedema.

Some patients in the HAE group did not have a diagnostic history specific to HAE (eg, abdominal pain, swelling, or edema) within the KUHP data. Their common symptoms might have been treated by their primary doctors or clinics and not at this university hospital. Furthermore, because HAE is a hereditary disorder, some patients may have been diagnosed through family testing. These factors appear to lead to a lower sensitivity score for the Japanese dataset than that for the US data.

The precision score was 23.6%, which is more than 14 times higher than that of the initial model. As mentioned in the Introduction section, only 20% of patients in Japan are diagnosed with HAE, which means that 80% of patients with HAE are undiagnosed. Therefore, the 211 patients from the control group who were suspected to have HAE in our model may be undiagnosed patients with HAE.

## Discussion

### Principal Findings

In this study, we developed an AI model for screening patients with HAE and validated its performance using 2 methods.

First, a large patient dataset was selected to build a model containing patient-level linked claims and EMR data from the United States. The advantage of this dataset is that it contains a long-term prescription and diagnostic history across multiple medical institutes. The diagnostic characteristics of patients with HAE were determined by analyzing the dataset. Based on these characteristics, we constructed a generalized linear model with regularization terms. At a threshold of 0.1, the sensitivity score was 52.5% and the precision score was 27.1% if patients with possible HAE were included in the correct answer group. When these were excluded from the correct answers, the sensitivity score was 61.5%.

We then applied this model to Japanese EMR data. This validation was conducted at a single university hospital using DWH data. Generally, patients often visit local hospitals and rarely visit university hospitals if they present with common symptoms. Considering this situation, data obtained from a single university may have some difficulty with model performance. Although the sensitivity score was lower than that of the US dataset (37.6%), the precision score reached 23.6% with a threshold value of 0.075. This implies that our model has a high possibility of identifying patients with undiagnosed HAE in Japan.

### Limitations

Our study had several limitations. Generally, because HAE is a rare disease, patient group data (correct answer data in machine learning) are quite small. In addition, the variance in each patient’s features was larger than that in common diseases. We also suggest possible limitations and countermeasures.

#### Family History

In our basic analysis of the HAE group, we found that some patients in the HAE group had a lower diagnostic history than others. We suspected that these patients had been diagnosed with HAE based on their family histories. Because our model uses the diagnostic history to calculate the probability, these cases are potentially difficult to detect.

#### US Patient Data Consists of Data From Multiple Hospitals

Our model may rely on the fact that US patient data consists of data from multiple hospitals. Collecting data from multiple hospitals will allow tracking of the records of a single patient across these hospitals and provide a more detailed medical history. For validation in the Japanese dataset, we could only use data from a single university hospital, which may be one of the reasons for the low sensitivity.

#### Potential Patients With HAE Might Be Included in the Control + D84.1 Groups

Since the HAE diagnosis rate was low, it is likely that there were more patients with HAE in the control + D84.1 groups. In our approach, we assigned the HAE group a prescription history of HAE drugs to keep the model conservative.

#### Possible Difference in Diagnostic Tendency Between the United States and Japan

If there are differences in how doctors make diagnostics between countries, we may need to customize the model or threshold to adapt it to Japan and other countries.

### Comparison With Previous Work

Few previous studies have focused on screening patients for rare diseases based on diagnostic histories such as medical claims. Nonetheless, some studies have focused on a few rare diseases. For example, a previous study used AI models based on diagnostic history to identify patients with Pompe disease [17]. In this study, 104 patients were flagged by the model to have the disease, but only 19 were determined by specialists to have a high likelihood of having Pompe disease, rendering a precision score of 18.27% [17]. In comparison, our model recorded a precision of 23.6%. Screening for rare diseases is extremely difficult compared with other common diseases, for which abundant data exist; however, our results indicate that AI models can show high performance for screening rare diseases.

### Conclusions and Future Directions

Considering the prevalence of HAE (1/50,000), the screening performance of this model was 1,000 times greater than that achieved through random searching using US data. Owing to their prevalence and recognition rates, identifying undiagnosed patients with rare diseases is an arduous task. This study suggests that patient screening for HAE may become significantly more efficient if this AI model is used. This approach is particularly valuable for the diagnosis and treatment of rare diseases.

In addition, during the validation phase using the Japanese data, the model was effective at a single university hospital. Although only the diagnosis codes recorded in the EMR were available, the model could detect patients with typical symptoms of HAE. The performance of the model can likely be improved further if this model is applied to the data from city hospitals or medical claims, which contain diagnostic histories of patients in multiple medical institutions. This can provide more comprehensive information on the symptoms and diagnostic histories of each patient.

In this study, only patients with a diagnostic history of HAE within the dataset were defined as correct answers. By providing a diagnosis rate, these data may include patients with undetected HAE. The model performance cannot be strictly calculated in such situations. Therefore, further studies are needed to determine whether patients with undiagnosed HAE should be included in the predicted group. This is because identifying undiagnosed patients with HAE is a critical issue, especially in Japan; we will implement a prospective clinical study using our AI model.

The constructed model may help researchers, physicians, and other health care professionals identify undiagnosed HAE cases. Eventually, if this strategy can identify undiagnosed patients and provide them with proper treatment, their quality of life will likely be improved.

## Appendix

Multimedia Appendix 1 Table of the explanatory variables.

## Abbreviations

AI: artificial intelligence
DISCOVERY: Diagnostic Consortium to Advance the Ecosystem for Hereditary Angioedema
DWH: data warehouse
EMR: electronic medical record
HAE: hereditary angioedema
ICD: International Classification of Diseases
KUHP: Kyoto University Hospital
MEDIS-DC: Medical Information System Development Center
ROC: receiver operating characteristic
