# Supplementary figures and images for: Early Diagnosis of Hereditary Angioedema in Japan Based on a US Medical Dataset: Algorithm Development and Validation
Source: JMIR Med Inform. 2024 Sep 13;12:e59858. doi: 10.2196/59858 (PMC11437219; doi:10.2196/59858)

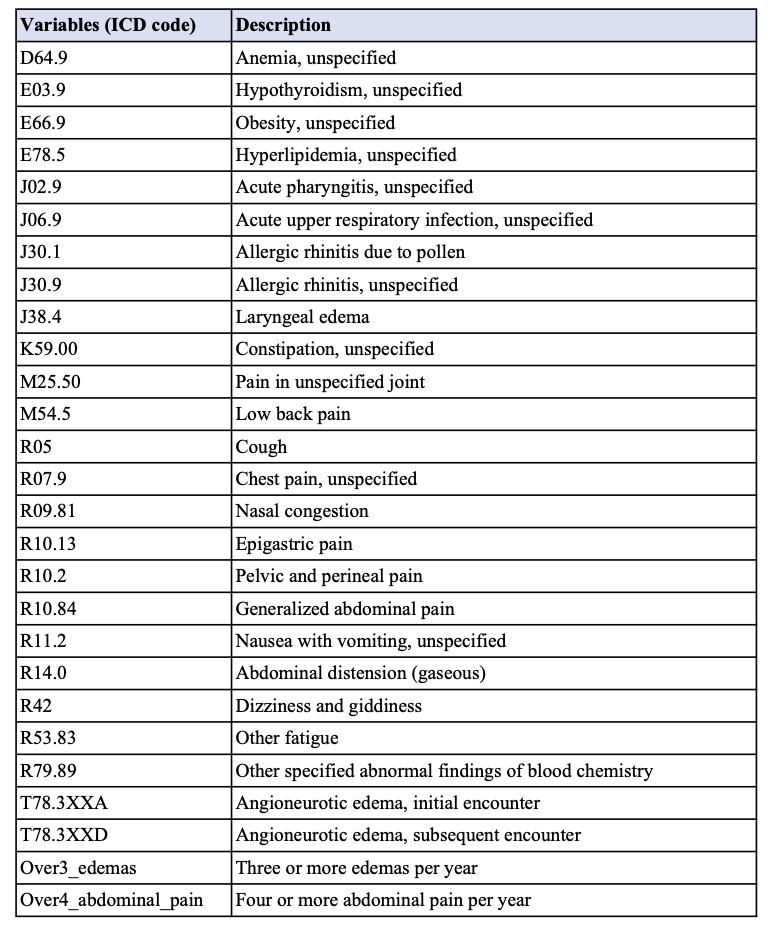

Supplement: Multimedia Appendix 1 [file medinform_v12i1e59858_app1.png]
